# [^18^F] fluoromisonidazole and [^18^F] fluorodeoxyglucose positron emission tomography in response evaluation after chemo-/radiotherapy of non-small-cell lung cancer: a feasibility study

**DOI:** 10.1186/1471-2407-6-51

**Published:** 2006-03-04

**Authors:** Bernd Gagel, Patrick Reinartz, Cengiz Demirel, Hans J Kaiser, Michael Zimny, Marc Piroth, Michael Pinkawa, Sven Stanzel, Branka Asadpour, Kurt Hamacher, Heinz H Coenen, Ulrich Buell, Michael J Eble

**Affiliations:** 1 Department of Radiotherapy, University of Aachen, Germany; 2 Department of Nuclear Medicine, University of Aachen, Germany; 3 Institute of Medical Statistics, University of Aachen, Germany; 4 Institute of Nuclear Chemistry, Research Center Juelich, Germany

## Abstract

**Background:**

Experimental and clinical evidence suggest that hypoxia in solid tumours reduces their sensitivity to conventional treatment modalities modulating response to ionizing radiation or chemotherapeutic agents. The aim of the present study was to show the feasibility of determining radiotherapeutically relevant hypoxia and early tumour response by ([^18^F] Fluoromisonidazole (FMISO) and [^18^F]-2-fluoro-2'-deoxyglucose (FDG) PET.

**Methods:**

Eight patients with non-small-cell lung cancer underwent PET scans. Tumour tissue oxygenation was measured with FMISO PET, whereas tumour glucose metabolism was measured with FDG PET. All PET studies were carried out with an ECAT EXACT 922/47^® ^scanner with an axial field of view of 16.2 cm. FMISO PET consisted of one static scan of the relevant region, performed 180 min after intravenous administration of the tracer. The acquisition and reconstruction parameters were as follows: 30 min emission scanning and 4 min transmission scanning with 68-Ge/68-Ga rod sources. The patients were treated with chemotherapy, consisting of 2 cycles of gemcitabine (1200 mg/m^2^) and vinorelbine (30 mg/m^2^) followed by concurrent radio- (2.0 Gy/d; total dose 66.0 Gy) and chemotherapy with gemcitabine (300–500 mg/m^2^) every two weeks. FMISO PET and FDG PET were performed in all patients 3 days before and 14 days after finishing chemotherapy.

**Results:**

FMISO PET allowed for the qualitative and quantitative definition of hypoxic sub-areas which may correspond to a localization of local recurrences. In addition, changes in FMISO and FDG PET measure the early response to therapy, and in this way, may predict freedom from disease, as well as overall survival.

**Conclusion:**

These preliminary results warrant validation in larger trials. If confirmed, several novel treatment strategies may be considered, including the early use of PET to evaluate the effectiveness of the selected therapy.

## Background

At present, combined modality treatment (chemotherapy followed by surgery or radio-/chemotherapy) for patients with locally advanced non-small-cell lung cancer (NSCLC) is being studied extensively. It is clear, however, that for different reasons, a substantial number of patients had lesser benefits from such intensive treatment. For example, tumour anaemia and tumour hypoxia are considered as multifactorial causes of tumour treatment resistance [4,21]. The causes of tumour hypoxia are multifactorial and include factors related to oxygen delivery, such as anaemia, abnormal tumour vasculature and blood flow, and the rate of oxygen consumption in tumours.

With positron emission tomography (PET), radio-labelled hypoxia-avid compounds can be applied to evaluate the oxygenation status in experimental or human tumours [2]. Fluorine-18 labelled Fluoromisonidazole [1-(2-nitro-1-imidazolyl)-2-hydroxy-3-fluoropropane, FMISO] is the most widely used nitroimidazole derivative in clinical PET, representing a non-invasive method for the quantification of the oxygenation status of subjacent tumours from PET data [7,19].

Conventional techniques used to monitor therapeutic effects in oncology, such as CT and MRI, are based on morphologic changes and show limited accuracy [11,14]. Several studies have demonstrated the ability of functional imaging techniques to detect subclinical alterations in tumour physiology and biochemistry resulting from efficacious therapy [1,6,13,15,18,20]. These alterations may occur long before a morphologic change in the tumour mass is apparent. PET with the glucose analogous fluorine-18fluorodeoxyglucose (FDG PET) allows non-invasive serial measurements of tumour glucose use. Previous studies have suggested that chemotherapy causes a measurable decrease in tumour glucose use within 1 to3 weeks after the commencement of therapy.

This study evaluates the impact and feasibility of determining tumour hypoxia by FMISO PET, and of one factor related to tumour metabolism, namely tumour glucose use, through FDG PET in patients with NSCLC in relation to response to radio-/chemotherapy.

## Methods

The Medical Ethical Committees of the University of Aachen approved the study. The study was conducted according to the Helsinki Declaration. All patients gave written informed consent before they were enrolled in the study.

### Patients and patient treatment

**Figure 1:**
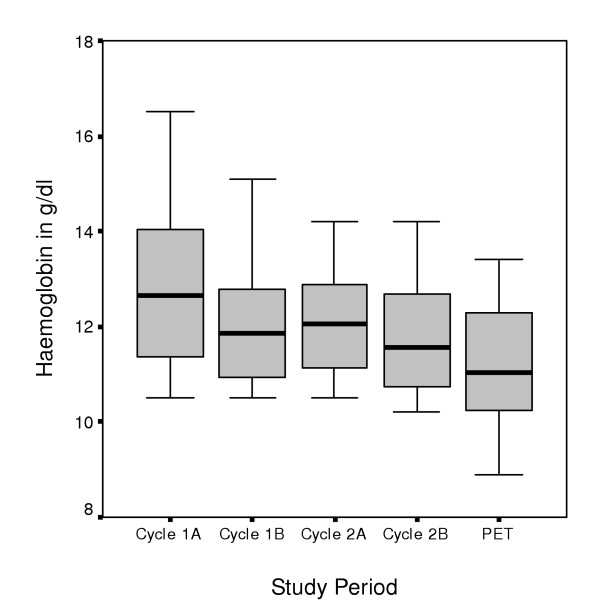
**Development in haemoglobin concentrations**. Change in haemoglobin concentrations during the course of chemotherapy. Mean difference in Hb concentration was 1.7 g/dl (SD = 0.4 g/dl) between baseline and final laboratory testing (paired T-test; p = 0.033)

All eight patients had histologically proven and unresectable NSCLC. The detailed patient characteristics are listed in Table 1. All patients were treated with chemotherapy consisting of 2 cycles of gemcitabine (1200 mg/m^2^) and vinorelbine (30 mg/m^2^) given on day 1, 8 (cycle 1A and 1B) and on day 22, 29 (cycle 2A and 2B). Gemcitabine was administered first in a 30-min i.v. infusion, followed by vinorelbine, which was given as a 5-min i.v. infusion. The change in haemoglobin concentrations during chemotherapy are shown in Figure 1.

Six patients were included in a phase I study to identify the dose-limiting toxicities (DLTs) and maximum tolerated dose (MTD) of gemcitabine when administered in a 14-day interval (three times) in combination with radiotherapy which began 14 days after the induction chemotherapy. Radiotherapy was administrated with 10- or 15-MV photons. The total radiation dose to the initial volume was 50.0 Gy in 2.0 Gy fractions over 5 weeks. A boost dose of 10 to 16 Gy to the gross tumour volume (defined by FDG PET after induction chemotherapy) was given in the same dose fractionation. The gemcitabine dose ranged between 300 and 500 mg/m^2 ^in 100 mg/m^2 ^increments. At the end of radio-/chemotherapy one of these patients developed a perpetual arrhythmia caused by hyperthyroidism, followed by a histologically proven atypical pneumonia caused by cytomegalovirus without leucopenia or neutropenia during the treatment. He died because of cardiopulmonary insufficiency.

One patient outside the phase I study suddenly died of a lung embolism after two cycles of chemotherapy, and one patient was initially excluded from the phase I study because of distant lymph node metastasis detected by FDG PET. The latter was being treated with two cycles of chemotherapy followed by radio-/chemotherapy, due to left primary bronchus compression. Following chemotherapy, one patient had decreased FDG- and FMISO uptakes in the primary tumour, whereas he had an increased FDG- and nearly unchanged FMISO-uptake in one large mediastinal lymph node metastasis. Here, both tumour localisations were evaluated separately as patient 7a (lymph node metastasis) and 7b (primary) (Tables 2 and 3). All patients were followed up for at least one year.

### Positron emission tomography

All PET studies were carried out with a Siemens ECAT EXACT 922/47 scanner with an axial field of 16.2 cm of view (Siemens CTI, Knoxville, TN). FMISO and FDG were produced at the Research Centre Juelich (Juelich, Germany). Quality assurance was done according to the GMP principles. FMISO PET and FDG PET were performed in all patients within 3 days before as well as 14 days after finishing induction chemotherapy. The relevant field of view was defined with the help of computed tomography. FMISO PET consisted of one static scan of the relevant region, performed 180 min after intravenous administration of 329 ± 36 MBq FMISO. The acquisition and reconstruction parameters were as follows: Acquisition consisted of 30 min emission scanning and 4 min transmission scanning with 68-Ge rod sources. The emission data were corrected for attenuation through the use of a segmented μ-map and empirical attenuation coefficients.

Following normalization and scatter correction, the emission scan was reconstructed with and without attenuation correction using a weighted iterative OSEM algorithm (ordered subsets-expectation maximization, 6 iterations, 16 substeps). In a final step, a three-dimensional isotropic Gauss-filter was applied (FWHM 8 mm). Transversal, coronal, and sagittal slices of 7 mm thickness were reconstructed with and without attenuation correction.

Three venous blood samples were taken at minute 0, 5, and 10 of the emission scans. The radioactivity concentrations were measured with a well counter and corrected for decay. An FDG PET of the tumour region was performed 60 ± 12 min after intravenous administration of 250 ± 60MBq FDG, using a whole-body acquisition protocol with 8 min emission scanning and 4 min transmission scanning. The data were also corrected for attenuation and reconstructed with the same parameters as for FMISO PET above.

For FMISO PET, tumour-to-muscle ratios of the regional radioactivity concentrations were calculated, using the transverse plane with the maximum FMISO uptake and manually drawn regions of interest of the tumour and the ipsilateral muscles. The tumour was defined according to the image data of FDG PET.

Mean and maximum standardized uptake values (SUV) of the FMISO and FDG uptake of the tumour were calculated after normalization of the radioactivity concentration to the injected radioactivity and the body weight. Additionally, the mean SUV was corrected for partial volume effects by applying recovery coefficients obtained from phantom studies [25].

Tumour response was evaluated by computed tomography (CT) of the chest and FGD PET (using visual assessment of response by tumour volume), according to standard WHO criteria [24].

## Results

Before and two weeks after chemotherapy treatment, mean and maximum standardized uptake values (SUV) of the FMISO and FDG as well as tumour-to-muscle ratios of FMISO were calculated for the tumour regions (Table 2). The metabolic responses to treatment are listed in Table 3.

Using FDG PET, the metabolic responses 2 weeks after completing induction chemotherapy (two cycles gemcitabine/vinorelbine) were as follows: All five patients with a decreased FDG- and FMISO uptake had a partial remission (PR) after chemotherapy. One patient died after chemotherapy, due to a lung embolism. Evaluating tumour response 6 weeks after radio-/chemotherapy three patients had a complete remission (CR), and one had a PR after radio-/chemotherapy. There was a discrepancy between FDG PET and CT response in one patient, who had a CR in FDG PET and PR in CT after 6 weeks, but had a CR in both methods of diagnostic examination after 18 weeks. One patient developed tumour oxygenation near to muscle oxygenation reaching CR after radio-/chemotherapy. There was a discrepancy between the FMISO and FDG PET in one patient with a decreased FMISO uptake. However, there was an increase in the mean SUV and a decreased maximum SUV in FDG PET. This patient had a local PR after induction chemotherapy and after radio-/chemotherapy, but developed histologically proven kidney metastasis, as well as an early local tumour progression. One patient showed a decrease in FMISO uptake, but a high increase in the FDG uptake. This patient reached a stable disease (SD) after chemotherapy and a local PR, but in the same way, disseminated lung metastases and a solitary cerebral metastasis after radio-/chemotherapy (Table 3).

**Figure 2:**
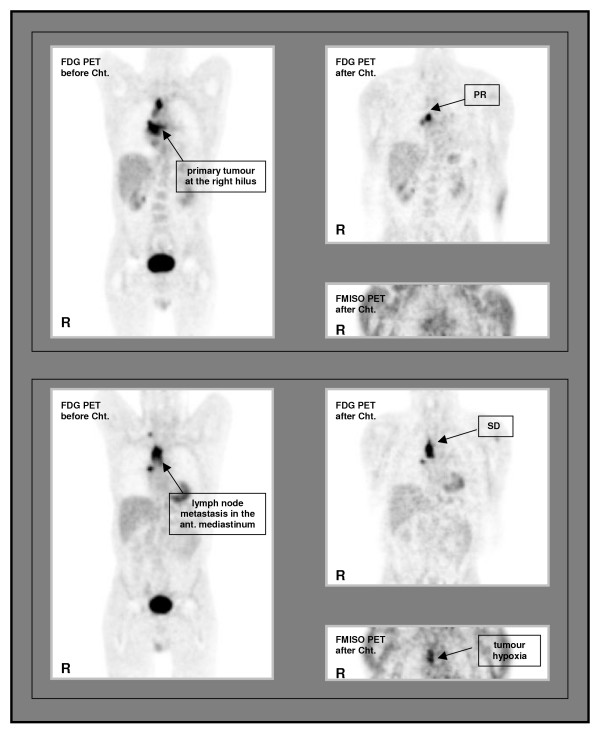
**Patient with differences in FMISO uptake**. Patient with different FMISO uptake in the primary tumour at the right hilus (coronar slices of the posterior mediastinum, anterior view of thorax) and in one large lymph node metastasis in the right mediastinum (coronar slices of the anterior mediastinum, anterior view of thorax) after chemotherapy: discrepant tumour response according to tumour oxygenation. Cht. = chemotherapy, SD = stable disease, PR = partial remission, R = right side

The patient (patient7a/b) who had different changes in FDG and FMISO uptakes in the primary tumour and in one large mediastinal lymph node metastasis after chemotherapy also showed a discrepant tumour response. Whereas there was a histologically proven CR (by bronchoscopy) of the primary tumour (decrease of FMISO and FDG uptake), there was no change in the extension of lymph node metastasis, where we found an increase of FDG values and only a small reduction in the FMISO tumour to muscle ratio with central accumulation of FMISO (Figure 2).

One patient with initial high FMISO uptake and decreased FMISO and FDG uptakes after chemotherapy showed a focal enhancement of FMISO at the chest wall, representing the localisation of early local recurrence.

There was no association between tumour hypoxia detected by FMISO PET and tumour glucose use measured by FDG PET.

## Discussion

A recent meta-analysis of 83 randomized clinical trials demonstrated a significant overall improvement in local control and survival with hypoxia modification [17]. Experimental and clinical evidence suggests that tumour hypoxia itself represents a prognostic factor which influences tumour growth, increasing malignant progression due to gene amplification and enhancing metastatic potential. In addition, the hypoxic fraction in solid tumours also reduces their sensitivity to conventional treatment modalities, thereby modulating the response to ionizing radiation and many chemotherapeutic agents [8-10,22,23], representing the most important treatment modalities in therapy of NSCLC.

It is recognized that nitroimidazoles bind selectively to hypoxic cells [16], leading to an intracellular accumulation of nitroimidazole. The FMISO uptake represents a global value for macroscopic tumour parts. As a non-invasive measure, this method is highly feasible for evaluating the state of oxygenation in subjacent tumours. In a prospective study we were able to validate FMISO PET for determination of radiotherapeutically relevant hypoxia by gold standard for measuring tissue oxygenation in human tumors, the computerized polarographic needle electrode system in patients with metastatic neck lymph node from a primary squamous carcinoma of the head and neck. High correlation was found between tumour-to-muscle ratio of FMISO and parameters of hypoxic fraction ≤ 2.5 mmHg and ≤ 5.0 mmHg [7]. Using dynamic and static FMISO PET scans, Eschmann et al could show that high ratios to reference tissues (mediastinum and muscle) correlated with the risk of relapse in NSCLC [5].

Our study was conducted in order to evaluate the feasibility of FMISO PET for the depiction of tumour oxygenation in NSCLC. Furthermore, we wanted to examine whether there is a reoxygenation after two-cycle chemotherapy, and whether there is an association between tumour metabolism or tumour hypoxia and tumour response or tumour recurrence. After chemotherapy, we were able to show a decrease in the FMISO uptake, reflecting reoxygenation in most patients, which resulted in a good tumour response. On the other hand, an increased (patient 6) or a nearly unchanged, high tumour to muscle ratio (patient 7a) corresponded to worse local tumour outcomes. But no association between initial high FMISO uptake and treatment outcome or tumour response could be detected.

Although there was a significant decrease in Hb concentrations due to chemotherapy (paired T-test; p = 0.033) influencing tumour oxygenation, we did not find a direct association between the reduction of the Hb concentration and FMISO or FDG uptake. Even the patient with an increased FMISO uptake showed stable Hb concentrations with an Hb of 13.4 g/dl at the time of second PET examinations (data not shown). Another patient showed a focal enhancement of FMISO at the chest wall after chemotherapy, representing the localisation of early local recurrence.

Mac Manus et al. prospectively studied the capacity of FDG PET and computed tomography (CT) to determine the response soon after radio- or radio-/chemotherapy in patients with NSCLC. Both CT and PET responses were individually significantly associated with survival duration; but in the multifactor analysis, this included the known prognostic factors of CT response, performance status, weight loss, and stage; only the PET response was significantly associated with the duration of survival (p < 0.0001) [12].

In another study, FDG PET imaging before and after induction therapy was prospectively evaluated in patients with oesophageal cancer to determine whether changes in PET images could measure the response to therapy. A comparison of the percentage decrease in SUV with the percentage of treatment effects through pathological examinations of oesophagectomy specimens indicated a correlation between large decreases in SUV and pathologic measurements of treatment effect [3].

In our study, all five patients with a clear decrease in the FDG uptake had a PR after chemotherapy; two had a PR and even three a CR after radio-chemotherapy. On the other hand, patients with nearly unchanged or increased FDG values showed worse tumour control or disease-free survival; therefore, changes in tumour metabolism detected by FDG PET may predict the tumour response and outcome in NSCLC.

## Conclusion

The results of this prospective study elucidate that in patients with NSCLC changes in FDG PET may represent early response to therapy, and in this way, may predict freedom from disease and overall survival. In addition a qualitative and quantitative definition of hypoxic sub-areas is possible on the basis of FMISO PET, and that these sub-areas may correspond to the localization of local recurrences, making the definition of a biological target volume possible. Nevertheless, data on the role of FMISO PET scans for NSCLC are limited. In addition, the problem of image-fusion and the low spatial resolution restrict the use for target definition. Our feasibility study included only 8 patients. Therefore, these preliminary results warrant validation in larger trials.

## Competing interests

Financial competing interests

The pharmaceutical company Hoffmann- La Roche partially financed the costs for FMISO, a substance used to carry out PET scans.

The authors declare that there are no other competing interests.

## Authors' contributions

BG has made substantial contributions to conception and design, acquisition of data, analysis and interpretation of data; PR has made substantial contributions to conception and design, acquisition of data, analysis and interpretation of data; CD has made substantial contributions to conception and design; HJK has been involved in acquisition of data, analysis and interpretation of data; MZ has made substantial contributions to conception and design, acquisition of data; MP has been involved in acquisition of data; MP has been involved in acquisition of data; SS has been involved in analysis and interpretation of data; BA has been involved in acquisition of data; KH has been involved in analysis and interpretation of data and has made contributions to conception and design; HHC has been involved in analysis and interpretation of data and has made contributions to conception and design; UB has been involved in analysis and interpretation of data and has made contributions to conception and design; MJE has been involved in analysis and interpretation of data and has made contributions to conception and design.

All authors read and approved the final manuscript.

## Pre-publication history

The pre-publication history for this paper can be accessed here:



## Figures and Tables

**Table 1:** Characteristics of the population

Characteristics	
Age	Years
Mean	58
Median	56
Range	41–76
Sex	No. of patients
Male	5
Female	3
ECOG performance scale	
0	4
1	4
Clinical stage	
IIIA	1
IIIB	6
IV	1
Histology	
Adenocarzinoma	3
Squamous carcinoma	4
Adenosquamous carcinoma	0
Large-cell carcinoma	1

ECOG performance Scale: 0 – normal activity; asymptomytic

1 – symptomatic; fully ambulatory

**Table 2:** Patient data of FDG and FMISO PET (*increase of FMISO uptake*; increase of mean SUV of FDG uptake)

**Pat.**	**FMISO**						**FDG**			
	
	**SUV mean **before/after Cht.		**SUV max. **before/after Cht.		**Tu./Musc. **before/after Cht.		**SUV mean **before/after Cht.		**SUV max **before/after Cht.	
1	1.90	1.40	2.12	1.83	1.12	1.04	9.70	7.70	12.32	8.16
2	2.20	1.40	2.48	1.63	1.45	1.26	17.10	8.10	20.19	9.69
3	1.90	1.90	2.57	2.43	1.54	1.36	14.10	7.10	16.28	7.96
4	3.70	2.20	4.59	2.43	5.85	1.51	14.00	8.30	19.02	9.40
**5**	**1.60**	**1.40**	**1.76**	**1.65**	**1.25**	**1.10**	**8.50**	**8.90**	**10.80**	**10.18**
*6*	*2.20*	*2.40*	*2.58*	*2.71*	*1.10*	*1.18*	*12.00*	*11.40*	*14.90*	*13.68*
**7a**	**2.60**	**2.00**	**3.02**	**2.28**	**1.86**	**1.75**	**8.60**	**10.60**	**11.85**	**11.99**
7b	2.60	2.00	3.02	2.34	1.86	1.52	8.60	5.10	11.85	7.15
**8**	**2.10**	**1.80**	**2.79**	**2.37**	**1.89**	**1.48**	**1.60**	**9.70**	**1.91**	**14.50**

Mean	2.31	1.83	2.77	2.19	1.99	1.36	10.47	8.54	13.23	10.30
SD	0.20	0.12	0.27	0.13	0.49	0.08	1.50	0.63	1.80	0.86

SUV = standard uptake value; Tu./Musc. = tumour to muscle ratio; Cht. = chemotherapy

**Table 3:** Differences in FDG and FMISO PET data before and after chemotherapy (*increase in FMISO uptake*; increase in mean SUV of FDG uptake); * kidney metastasis as well as an early local tumour progression; ^†^local PR but disseminated lung metastases.

**Pat.**	**FMISO**		**FDG**			
	
					**Local Tumour Response**	
					
	**SUV mean **post-prae Cht.	**Tu./Musc. **post-prae Cht.	**SUV mean **post-prae Cht.	**SUV max. **post-prae Cht.	post Cht.	post RT/Cht.
1	-0.50	-0.08	-2.00	-4,16	PR	CR
2	-0.80	-0.19	-9.00	-10.50	PR	
3	0.00	-0.14	-7.00	-8.32	PR	CR
4	-1.50	-4.34	-5.70	-9.62	PR	PR
**5**	**-0.20**	**-0.15**	**+0.40**	**-0.62**	**PR**	PR*
*6*	*+0.20*	*+0.18*	*-0.60*	*-1.22*	*SD*	*SD*
**7a**	**-0.60**	**-0.11**	**+2.00**	**+0.14**	**SD**	**SD**
7b	-0.60	-0.34	-3.50	-4.70	PR	PR
**8**	**-0.30**	**-0.41**	**+8.10**	**+12.59**	**SD**	**PR**^†^

Mean	-0.48	-0.62	-1.92	-2.93		
SD	0.17	0.47	1.73	2.34		

SUV = standard uptake value; Tu./Musc. = tumour to muscle ratio; Cht. = chemotherapy
